# Beyond mental disorders: The role of child maltreatment in childhood suicidal behaviour

**DOI:** 10.1177/00048674251413021

**Published:** 2026-02-09

**Authors:** Michelle L Townsend, Penelope Hasking, Glenn A Melvin, Rohan Borschmann

**Affiliations:** 1 School of Psychology, University of Wollongong, Wollongong, NSW, Australia; 2 School of Population Health, Curtin University, Perth, WA, Australia; 3 Curtin enAble Institute, Curtin University, Perth, WA, Australia; 4 SEED Lifespan, School of Psychology, Deakin University, Geelong, VIC, Australia; 5 Thames Valley Child and Adolescent Mental Health Services Tier 4 Provider Collaborative, Oxford Health NHS Foundation Trust, Oxfordshire, UK; 6 Department of Psychiatry, University of Oxford, Oxford, UK; 7 Centre for Adolescent Health, Murdoch Children’s Research Institute, Melbourne, VIC, Australia; 8 Justice Health Group, School of Population Health, Curtin University, Perth, WA, Australia; 9 Centre for Mental Health and Community Wellbeing, Melbourne School of Population and Global Health, University of Melbourne, Melbourne, VIC, Australia; 10 Melbourne School of Psychological Sciences, University of Melbourne, Melbourne, VIC, Australia

**Keywords:** Child abuse, child maltreatment, suicide, risk factors, mental disorders, primary prevention, children

## Abstract

Suicide is one of the leading causes of death in children aged 14 years and under in Australia, and child maltreatment is consistently identified as an antecedent. Despite this, not enough is known about the pathways from child maltreatment to suicidal behaviour, hampering prevention efforts. In this perspective, we examine the association between (1) various types of childhood maltreatment, and (2) the presence of mental disorders, and subsequent suicidal behaviours in children aged 14 years and under. We present a conceptual model of childhood suicidal behaviours which incorporates both direct and indirect mechanisms by which maltreatment (and other risk factors) exert an influence. Bodily intrusive maltreatment, especially sexual and physical abuse, significantly increases the risk of suicidal behaviours in childhood. Other forms of maltreatment, such as neglect and emotional abuse, may also contribute. While the presence of a mental disorder is another prominent risk factor for suicidal behaviours in adolescents and adults, there is less evidence of this association in childhood. Efforts to prevent child maltreatment can support suicide prevention efforts in children and other age groups. In addition, screening for suicidal behaviours and targeted interventions should be prioritised for populations at increased risk, particularly children with a history of maltreatment and their families.

Suicide among young people is a major global public health problem (Ward et al., 2021). The death of a child or adolescent by suicide has profound consequences for family, peers and the broader community (Soto-Sanz et al., 2019). One strong predictor of suicidal behaviours in children and adolescents is childhood maltreatment (Bruffaerts et al., 2010; Castellví et al., 2017). In this perspective, we argue that, in addition to the prevention of child maltreatment, there is an imperative to address the sequelae of child maltreatment within broader efforts to prevent childhood suicide. In Australia, suicide is the leading cause of death in children and young people aged 5–17 years (Australian Bureau of Statistics, 2019). The burden is disproportionately borne by Indigenous Australians, among whom the suicide rate is double that of their non-Indigenous peers (Australian Bureau of Statistics, 2019). Despite the magnitude of this problem, many factors associated with suicide remain poorly understood (Ati et al., 2021), with prediction models still close to chance after more than 50 years of international research (Franklin et al., 2017).

The scale of child maltreatment in Australia is substantial. The Australian Child Maltreatment Study of 8500 people aged 16 years and older found that 39.6% of respondents reported lifetime exposure to domestic violence, 32.0% reported experiences of physical abuse, 30.9% emotional abuse, 28.5% sexual abuse and 8.9% reported experiences of neglect during childhood (Mathews et al., 2023). These rates are considerably higher than the rate of young people under 18 years of age coming into contact with the Australian child protection system, which is currently recorded as one in every 32 children (Australian Institute of Health and Welfare, 2023). This discrepancy suggests that many of children who experience childhood maltreatment do not come to the attention of family support services or welfare authorities, which represents a missed opportunity to intervene (Moore et al., 2015). Differing thresholds between research measures and child protection notifications, and differences in definitions and measurements (Baldwin et al., 2019) across research and practice, may also contribute to the differences reported. Nonetheless, given the limited concordance between individuals reporting retrospectively and prospectively, these may also be different populations of people (Baldwin et al., 2019).

## Suicidal behaviours in those who have experienced child maltreatment

A 2020 global systematic review and meta-analysis examined the association between childhood maltreatment and suicidal behaviours among 337,185 children and adolescents (aged up to 24 years) (Angelakis et al., 2020). The authors reported that all forms of childhood maltreatment were associated with higher rates of suicide attempts and that sexual, physical and emotional abuse were each associated with an increased risk of suicidal ideation. However, the predominance of cross-sectional studies in this meta-analysis presents limitations, particularly in terms of causal inference and susceptibility to selection and reporting bias inherent in this study design. The Australian Child Maltreatment Study (Lawrence et al., 2023) also found that all forms of childhood maltreatment – including sexual, physical, and emotional abuse, neglect, and exposure to domestic violence – were associated with higher rates of suicide attempts in the past 12 months relative to those who did not experience child maltreatment. However, the low response rate (14%), likely recall bias, and cohort effects (participants ranged in age from 16 to over 65 years) may influence prevalence estimates and limit generalisations that can be made (Haslam et al., 2023). A recent study with university students across 18 countries (*n* = 72,288) also found childhood maltreatment elevated the risk for adolescents and young adults’ suicidal thoughts and behaviour (Mortier et al., 2025). These three large-scale studies (Angelakis et al., 2020; Lawrence et al., 2023; Mortier et al., 2025) provide evidence that individuals who have experienced child maltreatment are at increased risk of engaging in suicidal behaviours. A further large multi-country study (*N* = 55,299) with a representative sample aged 16 years and older (Bruffaerts et al., 2010), found that physical abuse, sexual abuse and neglect each increased the likelihood of a retrospectively reported suicide attempt in childhood (defined by the authors as aged 4–12 years) and also in adolescence (aged 13–19 years). The authors also reported that the influence of child maltreatment on suicide attempts (but not ideation) decreased in adulthood (Bruffaerts et al., 2010).

Although there is a substantial body of research linking childhood maltreatment to suicidal behaviours in adolescence and adulthood, there remains a significant gap in research specifically examining this relationship directly with children during childhood, with much of the knowledge drawn from retrospective self-reports. Research on adverse childhood experiences – including maltreatment – and suicide risk suggests that the likelihood of suicide attempts during childhood or adolescence stems from the temporal proximity of these experiences, the limited coping capacity of some children to deal with these stressors, and an inability to escape these situations (Dube et al., 2001). Together with other research (Coelho et al., 2016; Qi et al., 2014), these findings suggest that although experiencing childhood maltreatment may be one of the leading behavioural factors for suicide across all age groups (Australian Institute of Health Welfare, 2022), there may be differences in suicide behaviour risk profiles across the lifespan.

## Potential pathways between maltreatment and suicide

While the aetiology of suicidal behaviour in children remains poorly understood (Soto-Sanz et al., 2019), two possible pathways have been posited to account for why children (aged ⩽ 14 years) who have experienced maltreatment may be at increased risk of suicidal behaviours. First, there may be a direct effect related to the frequency and severity of the child maltreatment (Thompson et al., 2005). Some research has suggested that the dose-response relationship between child maltreatment and suicidal behaviours is stronger in the short-term than the medium- or long-term (Thompson et al., 2012), and further influenced by the victim’s age at the onset of the maltreatment (Dunn et al., 2013), severity of abuse and chronicity across developmental periods (Thompson et al., 2005). Other studies also support the hypothesised direct relationship between physical (Thompson et al., 2005) and sexual (Angelakis et al., 2020; Enns et al., 2006) abuse victimisation and subsequent suicidal ideation, in addition to suicide attempts in children and younger adolescents (Enns et al., 2006). This proposition is in keeping with a 2014 review of the theoretical, clinical and research evidence regarding the emergence of suicidal thoughts by O’Connor and Nock (2014), which suggested that the strength of the association between childhood adversities – including maltreatment – and suicidal behaviours decreases with age.

Second, there is a hypothesised pathway whereby childhood maltreatment has an indirect effect on suicidal behaviours via children’s identity formation and the subsequent development of mental health difficulties (Bruffaerts et al., 2010; Thompson et al., 2005). Violent and bodily intrusive maltreatment, such as physical and sexual abuse, may be uniquely related to suicidal behaviours, beyond other childhood adversities (Bruffaerts et al., 2010). It is hypothesised that physical and sexual maltreatment contributes to children developing a negative relationship with their physical bodies, and problematic development of their identity and psychological integrity, which may lead to later mental health difficulties, including suicidal behaviours (Bruffaerts et al., 2010).

The presence of mental disorders may also indirectly increase the risk of suicidal behaviours. While it is well established that child maltreatment increases the risk of developing a mental disorder over the lifespan (Negriff, 2020), there is ongoing debate regarding the extent to which the association between child maltreatment and suicidal behaviours are mediated by the presence of mental disorders (O’Connor and Nock, 2014), particularly in childhood. Bruffaerts et al. (2010) argue that the presence of childhood maltreatment (particularly intrusive and/or violent forms) is a stronger predictor of the onset and persistence of suicidal behaviours than the presence of a mental disorder. A study of 740 8-year-old children who had experienced maltreatment similarly reported that children’s experiences of maltreatment was a key risk factor in the development of suicidal ideation, although current child mental health functioning mediated the effects of the maltreatment (Thompson et al., 2005). These findings – of a potential direct relationship between violent and intrusive forms of maltreatment and suicidal behaviours, and an indirect relationship between other forms of abuse (e.g. neglect, emotional abuse) via the presence of a mental disorder – have also been substantiated in older populations (aged 15–69 years) (Sachs-Ericsson et al., 2017).

**Figure 1.**
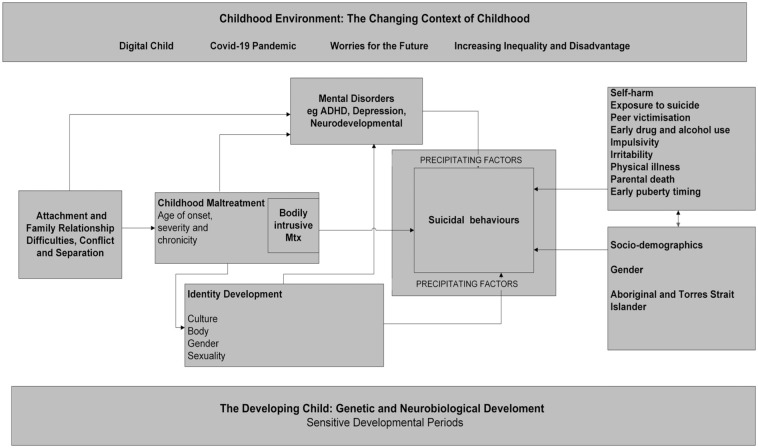
Conceptual framework: Direct and indirect pathways to childhood suicidal behaviours.

Figure 1 provides a conceptual understanding of childhood suicidal behaviours which incorporates both direct and indirect mechanisms by which maltreatment (and other risk factors) are related to suicide.

When accounting for these multifarious outcomes related to child maltreatment, several factors – including the age of onset, persistence, poly-victimisation and severity of maltreatment – may all contribute to subsequent suicidal behaviours (Bruffaerts et al., 2010; Thompson et al., 2005). These findings align with theories of suicidal behaviours informed by diathesis-stress processes, whereby early life vulnerabilities stemming from genetic and environmental factors interact with the stress resulting directly from maltreatment, leading to dysregulation in the hypothalamic–pituitary–adrenal axis, and may directly and indirectly contribute to subsequent suicidal behaviours (Bruffaerts et al., 2010; Castellví et al., 2017; O’Connor and Nock, 2014). Furthermore, maltreatment disrupts normative developmental processes, and a range of potential mechanisms – including cognitive, psychological, interpersonal and biological factors – are likely to act as mediators or moderators throughout children’s development. However, there has been limited investigation and little consensus regarding these mechanisms (Duprey et al., 2023).

There are several potential mechanistic pathways linking childhood maltreatment to suicidal behaviour in children that require further investigation. Understanding the influence of genetics and environment, viewed through a developmental lens (Cicchetti, 2016), is crucial to understanding suicidal behaviours in children. Key pathways between maltreatment and suicide warranting further investigation include neurobiological and epigenetic changes, dysregulation of stress response systems, disruptions in the attachment process and the development of maladaptive cognitions.

There is growing evidence that the timing of maltreatment exposure is critical, as experiences during sensitive developmental periods can lead to enduring brain changes which, in turn, can shape behavioural responses (Campbell, 2022). Maltreatment-related neurobiological adaptations may serve a protective function at one developmental phase, although later can become maladaptive as children progress through subsequent developmental stages (Teicher and Samson, 2016). An important area for further research is understanding how maltreatment alters neurobiological systems involved in threat detection and reward processing (Teicher and Samson, 2016), as these changes are implicated in suicidal behaviours (Campbell, 2022). Dysregulated stress responses also appear closely related with suicidal behaviour. Oquendo et al. (2014) argues that the corticotropin-releasing hormone/hypothalamic–pituitary–adrenal axis (CRH-HPA) system plays a key role in suicide vulnerability. This aligns with Bruffaerts et al. (2010), who suggest intrusive childhood maltreatment amplifies HPA axis sensitivity, increasing children’s vulnerability to suicidal behaviours. Early adversity may also trigger epigenetic and inflammatory changes that further compound risk for suicidal behaviours (Baldini et al., 2025; Brodsky, 2016), although further research is required in child populations (Kim et al., 2014).

Maltreated children often experience disruptions to the development of secure attachments with their caregivers (Baer and Martinez, 2006), leading to difficulties in emotion regulation, coping mechanisms and interpersonal relationships (Szeifert et al., 2025). Non-secure attachment has been found to directly affect suicidal behaviour in children, and it has been posited that insecure attachment with caregivers also flows on to children’s relationships with others, including peers (Silva Filho et al., 2023). This, in turn, may contribute to thwarted belongingness and disconnection from others (Handley et al., 2019). This aligns with Joiner’s (2005) Interpersonal-Psychological Theory of Suicidal Behaviour. In keeping with this theory, experiencing violence and/or witnessing violence perpetrated against others may also increase an individual’s capability for suicide (Handley et al., 2019). Thompson et al. (2005) similarly found that experiencing or witnessing violence was a key risk factor in the development of suicidal ideation. Maltreatment can foster maladaptive cognitive schemas such as self-blame, worthlessness and hopelessness, as well as impairing emotional and affect regulation (Yiğit et al., 2021), which may contribute to suicidal behaviour (Szeifert et al., 2025). There may also be some gender differences in how children process and understand their maltreatment experiences (Toth and Cicchetti, 2013) and, to date, limited research has sought to explore this. Together, these findings highlight the importance of incorporating biomarkers alongside psychosocial assessment in future research (Johnston et al., 2022), particularly in longitudinal designs across key developmental periods (Cicchetti, 2016).

## Implications

Efforts to improve the prediction of suicide have not reduced the number of children and adolescents dying by suicide (Glenn and Nock, 2014). In Australia, it is estimated that childhood maltreatment accounts for approximately 41% of suicide attempts across the lifespan (Grummitt et al., 2024). Given the strong link between childhood maltreatment and suicide risk, effective suicide prevention efforts must include strategies to address childhood maltreatment. It has been posited that effective interventions to prevent child sexual abuse could reduce suicide attempts in young people aged 12–26 years by 14.3% (Castellví et al., 2017) (population attributable risk estimate based on a meta-analysis of 34 studies). A comprehensive, multi-tiered approach is essential, combining universal, targeted and indicated interventions to prevent and respond to child maltreatment. Given the widespread occurrence of child maltreatment and its well-documented physical and psychological health risks, including mental disorders and suicidal behaviours (Baldwin et al., 2023), universal approaches to preventing maltreatment would likely improve population health outcomes (Lawrence et al., 2023). These universal approaches encompass the provision of early support to families before difficulties (or maltreatment) occurs, as well as interventions aimed at reducing childhood maltreatment (Branco et al., 2022; Sahle et al., 2022). Evidence indicates that universal parenting programmes can effectively prevent and reduce child maltreatment, as well as strengthen positive parenting (Branco et al., 2022; Coore Desai et al., 2017). Emerging evidence suggests that systemic change may be occurring as a result of public health initiatives, with the Australian Child Maltreatment Study reporting a decrease in reports of physical and sexual abuse among younger individuals in the study cohort (aged 16–24 years) in comparison to older age group cohorts, potentially reflecting the increased awareness and education in the community (Mathews et al., 2023). Furthermore, given (a) the proportion of the population affected and (b) the knowledge that a large proportion of children experiencing maltreatment will not come to the awareness of child protection agencies, universal interventions to strengthen children’s coping mechanisms and emotional regulation will also likely be of benefit (Lawrence et al., 2023). Carpendale et al.’s (2025) study of Australian year 6 students (*n* = 18,643) demonstrated that the universal implementation of evidence-based social-emotional learning programmes that incorporate opportunities for skill instruction and practice significantly promotes children’s socio-emotional functioning and overall well-being.

Furthermore, targeted interventions should be prioritised for populations at increased risk, particularly children with a history of maltreatment and their families. Screening for suicidal behaviours is crucial in children with identified mental health issues (Kovess-Masfety et al., 2015) and those who have experienced child maltreatment (O’Hare et al., 2023). It can be difficult to identify in real-time children who are being maltreated; therefore, screening for suicidal behaviours should occur when there is the presence of other risk factors for childhood maltreatment, for example, special healthcare needs or disabilities, family poverty, parental mental health disorder, parental substance use disorder and/or parental intimate partner violence (Austin et al., 2020). Child maltreatment is likely to indicate the presence of other challenges for families (e.g. parenting stress and dysfunction, social isolation and intergenerational maltreatment experiences; Brown et al., 1999); as such, addressing child and parent mental disorders, and social disadvantage, and enhancing family well-being is a vital component of any effective intervention.

Tertiary clinical implications include a focus on suicide prevention and intervention for children who have experienced maltreatment. Clinicians utilising trauma-informed care approaches could target identity formation and mental illness to ameliorate the effects of maltreatment, thereby preventing suicidal behaviours for some children and young people (Angelakis et al., 2020). Receiving care from a clinician has also been found to be a protective factor for suicidal behaviours among children who have experienced sexual abuse (Angelakis et al., 2020).

Future research requires longitudinal study designs (Enns et al., 2006) that elucidate the link between the types of child maltreatment and differential suicidal outcomes (Angelakis et al., 2020), particularly the potential mechanisms involved. An improved understanding of how children transition from suicidal ideation to planning and then attempt is also urgently needed. An important research question relates to understanding the role of the timing, severity and chronicity of maltreatment on the onset, persistence and transition of suicidal behaviours across the lifespan. The triangulation of data from multiple sources, where possible, will offer more comprehensive estimates and understandings (Bull et al., 2025). It is imperative that we improve our understanding of the various drivers of child maltreatment and enhance our efforts to reduce the prevalence in the community. Preventing childhood maltreatment may represent one of the most effective early interventions for reducing suicide risk.

## References

[bibr1-00048674251413021] Angelakis I Austin JL Gooding P (2020) Association of childhood maltreatment with suicide behaviors among young people: A systematic review and meta-analysis. JAMA Network Open 3: e2012563. 10.1001/jamanetworkopen.2020.12563 PMC7407092 32756929

[bibr2-00048674251413021] Ati NAL Paraswati MD Windarwati HD (2021) What are the risk factors and protective factors of suicidal behavior in adolescents? A systematic review. Journal of Child and Adolescent Psychiatric Nursing 34: 7–18. 33025698 10.1111/jcap.12295

[bibr3-00048674251413021] Austin AE Lesak AM Shanahan ME (2020) Risk and protective factors for child maltreatment: A review. Current Epidemiology Reports 7(4): 334–342. 34141519 10.1007/s40471-020-00252-3 PMC8205446

[bibr4-00048674251413021] Australian Bureau of Statistics (2019) Causes of Death, Australia, 2018. Canberra, ACT, Australia: Australian Bureau of Statistics.

[bibr5-00048674251413021] Australian Institute of Health and Welfare (2023) Child protection Australia 2021–22. Canberra, ACT, Australia: AIHW.

[bibr6-00048674251413021] Australian Institute of Health Welfare (2022) Australian Burden of Disease Study 2022. Canberra, ACT, Australia: AIHW.

[bibr7-00048674251413021] Baer JC Martinez CD (2006) Child maltreatment and insecure attachment: A meta-analysis. Journal of Reproductive and Infant Psychology 24: 187–197.

[bibr8-00048674251413021] Baldini V Gnazzo M Varallo G , et al. (2025) Inflammatory markers and suicidal behavior: A comprehensive review of emerging evidence. Annals of General Psychiatry 24: 36. 40442662 10.1186/s12991-025-00575-9 PMC12124015

[bibr9-00048674251413021] Baldwin JR Reuben A Newbury JB , et al. (2019) Agreement between prospective and retrospective measures of childhood maltreatment: A systematic review and meta-analysis. JAMA Psychiatry 76: 584–593. 30892562 10.1001/jamapsychiatry.2019.0097 PMC6551848

[bibr10-00048674251413021] Baldwin JR Wang B Karwatowska L , et al. (2023) Childhood maltreatment and mental health problems: A systematic review and meta-analysis of quasi-experimental studies. American Journal of Psychiatry 180: 117–126. 36628513 10.1176/appi.ajp.20220174 PMC7614155

[bibr11-00048674251413021] Branco MSS Altafim ERP Linhares MBM (2022) Universal intervention to strengthen parenting and prevent child maltreatment: Updated systematic review. Trauma, Violence & Abuse 23: 1658–1676. 10.1177/15248380211013131 33973499

[bibr12-00048674251413021] Brodsky BS (2016) Early childhood environment and genetic interactions: The diathesis for suicidal behavior. Current Psychiatry Reports 18: 86. 27484207 10.1007/s11920-016-0716-z

[bibr13-00048674251413021] Brown J Cohen P Johnson JG , et al. (1999) Childhood abuse and neglect: Specificity of effects on adolescent and young adult depression and suicidality. Journal of the American Academy of Child and Adolescent Psychiatry 38: 1490–1496. 10596248 10.1097/00004583-199912000-00009

[bibr14-00048674251413021] Bruffaerts R Demyttenaere K Borges G , et al. (2010) Childhood adversities as risk factors for onset and persistence of suicidal behaviour. The British Journal of Psychiatry 197: 20–27. 20592429 10.1192/bjp.bp.109.074716 PMC2894980

[bibr15-00048674251413021] Bull C Trott M Kisely S (2025) Addressing the challenges of child maltreatment measurement: Examining the types of data we use and how we use them. Australian and New Zealand Journal of Psychiatry 59: 301–303. 39991901 10.1177/00048674251320308

[bibr16-00048674251413021] Campbell KA (2022) The neurobiology of childhood trauma, from early physical pain onwards: As relevant as ever in today’s fractured world. European Journal of Psychotraumatology 13: 2131969. 36276555 10.1080/20008066.2022.2131969 PMC9586666

[bibr17-00048674251413021] Carpendale EJ Green MJ Dix KL , et al. (2025) An exploratory evaluation of universal social-emotional learning programs delivered during elementary school to Australian students. Journal of School Psychology 110: 101447. 10.1016/j.jsp.2025.101447 40506177

[bibr18-00048674251413021] Castellví P Miranda-Mendizábal A Parés-Badell O , et al. (2017) Exposure to violence, a risk for suicide in youths and young adults. A meta-analysis of longitudinal studies. Acta Psychiatrica Scandinavica 135: 195–211. 27995627 10.1111/acps.12679

[bibr19-00048674251413021] Cicchetti D (2016) Socioemotional, personality, and biological development: Illustrations from a multilevel developmental psychopathology perspective on child maltreatment. Annual Review of Psychology 67: 187–211. 10.1146/annurev-psych-122414-033259 26726964

[bibr20-00048674251413021] Coelho BM Andrade LH Borges G , et al. (2016) Do childhood adversities predict suicidality? Findings from the general population of the metropolitan area of Sao Paulo, Brazil. PLoS One 11: e0155639. 10.1371/journal.pone.0155639 PMC4871559 27192171

[bibr21-00048674251413021] Coore Desai C Reece JA Shakespeare-Pellington S (2017) The prevention of violence in childhood through parenting programmes: A global review. Psychology, Health & Medicine 22: 166–186. 10.1080/13548506.2016.1271952 28133982

[bibr22-00048674251413021] Dube SR Anda RF Felitti VJ , et al. (2001) Childhood abuse, household dysfunction, and the risk of attempted suicide throughout the life span: Findings from the adverse childhood experiences study. Journal of the American Medical Association 286: 3089–3096. 11754674 10.1001/jama.286.24.3089

[bibr23-00048674251413021] Dunn EC McLaughlin KA Slopen N , et al. (2013) Developmental timing of child maltreatment and symptoms of depression and suicidal ideation in young adulthood: Results from the national longitudinal study of adolescent health. Depression and Anxiety 30: 955–964. 23592532 10.1002/da.22102 PMC3873604

[bibr24-00048674251413021] Duprey EB Handley ED Wyman PA , et al. (2023) Child maltreatment and youth suicide risk: A developmental conceptual model and implications for suicide prevention. Development and Psychopathology 35: 1732–1755. 36097812 10.1017/S0954579422000414 PMC10008764

[bibr25-00048674251413021] Enns MW Cox BJ Afifi TO , et al. (2006) Childhood adversities and risk for suicidal ideation and attempts: A longitudinal population-based study. Psychological Medicine 36: 1769–1778. 16999880 10.1017/S0033291706008646

[bibr26-00048674251413021] Franklin JC Ribeiro JD Fox KR , et al. (2017) Risk factors for suicidal thoughts and behaviors: A meta-analysis of 50 years of research. Psychological Bulletin 143: 187–232. 27841450 10.1037/bul0000084

[bibr27-00048674251413021] Glenn CR Nock MK (2014) Improving the prediction of suicidal behavior in youth. International Journal of Behavioral and Consultation Therapy 9: 7–10. 29924107 PMC4557617

[bibr28-00048674251413021] Grummitt L Baldwin JR Lafoa’i J , et al. (2024) Burden of mental disorders and suicide attributable to childhood maltreatment. JAMA Psychiatry 81: 782–788. 38717764 10.1001/jamapsychiatry.2024.0804 PMC11079790

[bibr29-00048674251413021] Handley ED Warmingham JM Rogosch FA , et al. (2019) Infancy onset maltreatment and the development of suicide ideation: An investigation of moderation by oxytocin-related gene polymorphisms. Journal of Affective Disorders 257: 421–427. 31306993 10.1016/j.jad.2019.06.051 PMC6711826

[bibr30-00048674251413021] Haslam DM Lawrence DM Mathews B , et al. (2023) The Australian Child Maltreatment Study (ACMS), a national survey of the prevalence of child maltreatment and its correlates: Methodology. Medical Journal of Australia 218: S5–S12. 10.5694/mja2.51869 PMC10953333 37004182

[bibr31-00048674251413021] Johnston JN Campbell D Caruncho HJ , et al. (2022) Suicide biomarkers to predict risk, classify diagnostic subtypes, and identify novel therapeutic targets: 5 years of promising research. International Journal of Neuropsychopharmacology 25: 197–214. 34865007 10.1093/ijnp/pyab083 PMC8929755

[bibr32-00048674251413021] Joiner T (2005) Why People Die by Suicide. Cambridge, MA: Harvard University Press.

[bibr33-00048674251413021] Kim JW Szigethy EM Melhem NM , et al. (2014) Inflammatory markers and the pathogenesis of pediatric depression and suicide: A systematic review of the literature. The Journal of Clinical Psychiatry 75: 1242–1253. 25470085 10.4088/JCP.13r08898

[bibr34-00048674251413021] Kovess-Masfety V Pilowsky DJ Goelitz D , et al. (2015) Suicidal ideation and mental health disorders in young school children across Europe. Journal of Affective Disorders 177: 28–35. 25745832 10.1016/j.jad.2015.02.008

[bibr35-00048674251413021] Lawrence DM Hunt A Mathews B , et al. (2023) The association between child maltreatment and health risk behaviours and conditions throughout life in the Australian child maltreatment study. Medical Journal of Australia 218: S34–S39. 10.5694/mja2.51877 PMC10952518 37004181

[bibr36-00048674251413021] Mathews B Pacella R Scott JG , et al. (2023) The prevalence of child maltreatment in Australia: Findings from a national survey. Medical Journal of Australia 218: S13–S18. 10.5694/mja2.51873 PMC10953347 37004184

[bibr37-00048674251413021] Moore SE Scott JG Ferrari AJ , et al. (2015) Burden attributable to child maltreatment in Australia. Child Abuse & Neglect 48: 208–220. 26056058 10.1016/j.chiabu.2015.05.006

[bibr38-00048674251413021] Mortier P Yang X Altwaijri YA , et al. (2025) The associations of childhood adversities and mental disorders with suicidal thoughts and behaviors – Results from the world mental health international college student initiative. Psychiatry Research 350: 116555. 40450963 10.1016/j.psychres.2025.116555 PMC12240005

[bibr39-00048674251413021] Negriff S (2020) ACEs are not equal: Examining the relative impact of household dysfunction versus childhood maltreatment on mental health in adolescence. Social Science & Medicine 245: 112696. 31785426 10.1016/j.socscimed.2019.112696 PMC6961803

[bibr40-00048674251413021] O’connor RC Nock MK (2014) The psychology of suicidal behaviour. The Lancet Psychiatry 1: 73–85. 26360404 10.1016/S2215-0366(14)70222-6

[bibr41-00048674251413021] O’Hare K Watkeys O Harris F , et al. (2023) Self-harm and suicidal ideation in children and adolescents in contact with child protection services. Medical Journal of Australia 218: 526–527. 36970987 10.5694/mja2.51898 PMC10952315

[bibr42-00048674251413021] Oquendo MA Sullivan GM Sudol K , et al. (2014) Toward a biosignature for suicide. American Journal of Psychiatry 171: 1259–1277. 25263730 10.1176/appi.ajp.2014.14020194 PMC4356635

[bibr43-00048674251413021] Qi X Hu W Page A , et al. (2014) Dynamic pattern of suicide in Australia, 1986-2005: A descriptive-analytic study. BMJ Open 4: e005311. 10.1136/bmjopen-2014-005311 PMC4120400 25079935

[bibr44-00048674251413021] Sachs-Ericsson NJ Stanley IH Sheffler JL , et al. (2017) Non-violent and violent forms of childhood abuse in the prediction of suicide attempts: Direct or indirect effects through psychiatric disorders? Journal of Affective Disorders 215: 15–22. 28292658 10.1016/j.jad.2017.03.030

[bibr45-00048674251413021] Sahle BW Reavley NJ Morgan AJ , et al. (2022) A Delphi study to identify intervention priorities to prevent the occurrence and reduce the impact of adverse childhood experiences. Australian and New Zealand Journal of Psychiatry 56: 686–694. 34231407 10.1177/00048674211025717

[bibr46-00048674251413021] Silva Filho OCD Avanci JQ Pires TO , et al. (2023) Attachment, suicidal behavior, and self-harm in childhood and adolescence: A study of a cohort of Brazilian schoolchildren. BMC Pediatrics 23: 403. 37592202 10.1186/s12887-023-04215-7 PMC10433545

[bibr47-00048674251413021] Soto-Sanz V Castellví P Piqueras JA , et al. (2019) Internalizing and externalizing symptoms and suicidal behaviour in young people: A systematic review and meta-analysis of longitudinal studies. Acta Psychiatrica Scandinavica 140: 5–19. 30980525 10.1111/acps.13036

[bibr48-00048674251413021] Szeifert NM Oláh B Gonda X (2025) The mediating role of adult attachment styles between early traumas and suicidal behaviour. Scientific Reports 15: 15855. 40328875 10.1038/s41598-025-00831-8 PMC12056111

[bibr49-00048674251413021] Teicher MH Samson JA (2016) Annual research review: Enduring neurobiological effects of childhood abuse and neglect. Journal of Child Psychology and Psychiatry 57: 241–266. 26831814 10.1111/jcpp.12507 PMC4760853

[bibr50-00048674251413021] Thompson R Briggs E English DJ , et al. (2005) Suicidal ideation among 8-year-olds who are maltreated and at risk: Findings from the LONGSCAN studies. Child Maltreatment 10: 26–36. 15611324 10.1177/1077559504271271

[bibr51-00048674251413021] Thompson R Litrownik AJ Isbell P , et al. (2012) Adverse experiences and suicidal ideation in adolescence: Exploring the link using the LONGSCAN samples. Psychology of Violence 2: 211. 10.1037/a0027107 PMC3857611 24349862

[bibr52-00048674251413021] Toth SL Cicchetti D (2013) A developmental psychopathology perspective on child maltreatment. Child Maltreatment 18: 135–139. 23886641 10.1177/1077559513500380 PMC4520222

[bibr53-00048674251413021] Ward JL Azzopardi PS Francis KL , et al. (2021) Global, regional, and national mortality among young people aged 10–24 years, 1950–2019: A systematic analysis for the global burden of disease study 2019. The Lancet 398: 1593–1618. 10.1016/S0140-6736(21)01546-4 PMC8576274 34755628

[bibr54-00048674251413021] Yiğit I Kiliç H Guzey Yiğit M , et al. (2021) Emotional and physical maltreatment, early maladaptive schemas, and internalizing disorders in adolescents: A multi-group path model of clinical and non-clinical samples. Current Psychology 40: 1356–1366.

